# Supplementary figures and images for: Ketoacid Analogues Supplementation in Chronic Kidney Disease and Future Perspectives
Source: Nutrients. 2019 Sep 3;11(9):2071. doi: 10.3390/nu11092071 (PMC6770434; doi:10.3390/nu11092071)

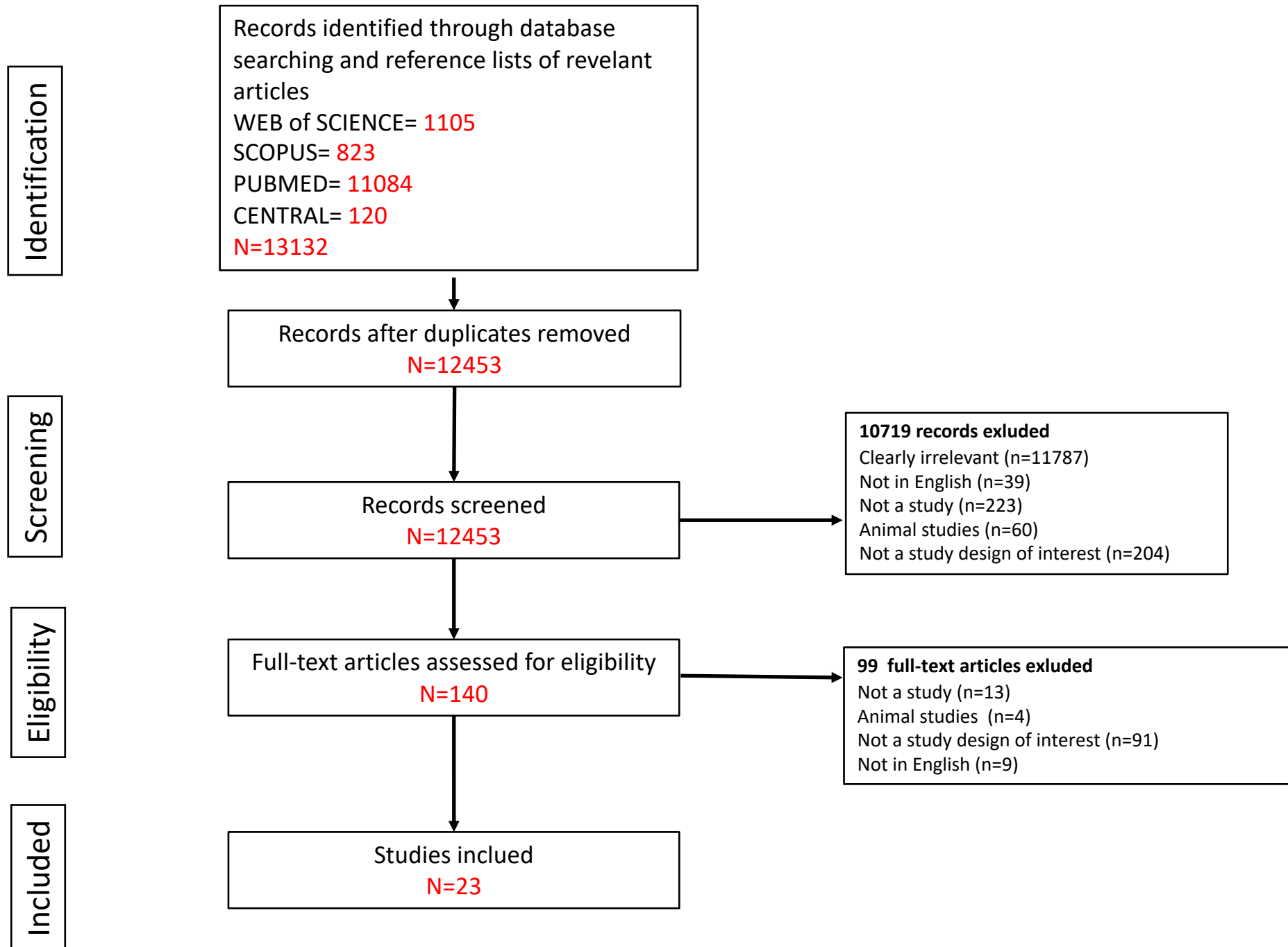

Figure S1. Identification process for randomized clinical studies.

Supplement: Supplementary file 1 [file nutrients-11-02071-s001.pdf]
